# Plant–Entomopathogenic Fungi Interaction: Recent Progress and Future Prospects on Endophytism-Mediated Growth Promotion and Biocontrol

**DOI:** 10.3390/plants13101420

**Published:** 2024-05-20

**Authors:** S. M. Ahsan, Md. Injamum-Ul-Hoque, Ashim Kumar Das, Md. Mezanur Rahman, Md. Mahi Imam Mollah, Narayan Chandra Paul, Hyong Woo Choi

**Affiliations:** 1 Department of Plant Medicals, Andong National University, Andong 36729, Republic of Korea; smvahsan@gmail.com; 2 Department of Applied Biosciences, Kyungpook National University, Daegu 41566, Republic of Korea; injamumrassel@gmail.com (M.I.-U.-H.); ashim@knu.ac.kr (A.K.D.); 3 Department of Plant and Soil Science, Institute of Genomics for Crop Abiotic Stress Tolerance, Texas Tech University, Lubbock, TX 79409, USA; mrahman@bsmrau.edu.bd; 4 Department of Entomology, Patuakhali Science and Technology University, Dumki, Patuakhali 8602, Bangladesh; mahiimam@pstu.ac.bd; 5 Kumho Life Science Laboratory, Chonnam National University, Gwangju 61186, Republic of Korea; ncpaulcnu@gmail.com; 6 Institute of Cannabis Biotechnology, Andong National University, Andong 36729, Republic of Korea

**Keywords:** biocontrol, endophyte, entomopathogenic fungi, growth promotion, interaction

## Abstract

Entomopathogenic fungi, often acknowledged primarily for their insecticidal properties, fulfill diverse roles within ecosystems. These roles encompass endophytism, antagonism against plant diseases, promotion of the growth of plants, and inhabitation of the rhizosphere, occurring both naturally and upon artificial inoculation, as substantiated by a growing body of contemporary research. Numerous studies have highlighted the beneficial aspects of endophytic colonization. This review aims to systematically organize information concerning the direct (nutrient acquisition and production of phytohormones) and indirect (resistance induction, antibiotic and secondary metabolite production, siderophore production, and mitigation of abiotic and biotic stresses) implications of endophytic colonization. Furthermore, a thorough discussion of these mechanisms is provided. Several challenges, including isolation complexities, classification of novel strains, and the impact of terrestrial location, vegetation type, and anthropogenic reluctance to use fungal entomopathogens, have been recognized as hurdles. However, recent advancements in biotechnology within microbial research hold promising solutions to many of these challenges. Ultimately, the current constraints delineate potential future avenues for leveraging endophytic fungal entomopathogens as dual microbial control agents.

## 1. Introduction

The entomopathogenic fungi (EPF) are pathogenic microbes which invade and kill arthropods. They are members of different taxonomic classes, which include Oomycetes, Chytridiomycota, Microsporidia, Entomophthoromycota, Basidiomycota, and Ascomycota, and comprise about 100 genera and 700 species. The key pathogens that cause EPF-mediated death are Zygomycota, Basidiomycota, Deuteromycota, and Ascomycota, as well as Microsporidia. The next stage of the life cycle begins when the spores of the EPF come in contact with the host tissue. The spores then germinate and penetrate the insect cuticle, and colonize the inner layers, causing the death of the insect [1]. The appressorium, resembling a ‘mechanically pressing’ virulent structure, is formed at the end of the germ tube. It secretes cuticle-degrading enzymes that assist the fungus in breaching the arthropod host’s integument. Furthermore, these fungi are capable of endophytic colonization, often observed in plant roots and, on occasion, throughout various above-ground plant tissues.

Endophytes, a class of tissue-dwelling microorganisms found in all plant species, often do not cause visible symptoms of disease. Some possess the ability to move internally within plant tissues, colonizing roots, stems, bark, and foliage. They utilize vertical channels from plants to seeds or scatter asexual or sexual spores horizontally to spread within uninfected plants. Endophytes are essential for plant growth and stress tolerance. Endophytic insect pathogenic fungi (EIPF) play a vital role in enhancing plant resilience to various stresses and promoting soil nutrient distribution. They achieve this by colonizing the roots and providing support against pathogens, herbivorous pests, salinity, and drought [2,3]. The order Hypocreales within the phylum Ascomycota is renowned for harboring EIPF [4]. Prominent examples of hypocrealean fungi include *Beauveria*, *Hirsutella*, *Isaria*, *Metarhizium*, *Verticillium*/*Lecanicillium*, *Nomuraea*, and *Cordyceps* (*Paecilomyces*), which are well-known as EIPF [1,3,5,6]. Only a few studies have thoroughly investigated the emerging role of endophytic fungal entomopathogens as enhancers of plant growth and biocontrol agents. Grasping the complete potential of interactions between plants and fungal entomopathogens could pave the way for more effective utilization of these fungi in biocontrol strategies and could open up new avenues for their varied applications in the future [2,7]. This review concentrates on the key themes of rhizosphere colonization, the signaling factor exchange between plants and fungi, the modulation of plant defense responses, the nutrient trafficking dynamics, and the tripartite interactions involving insects and other microorganisms. Data on the underlying mechanisms which are involved in the interaction of plant and entomopathogenic fungi (EPF) are yet to be characterized. This review is not only a very detailed account of the interactions described but it also addresses the gaps that need to be explored further. Additionally, we outline current limitations and propose future research directions aimed at leveraging endophytic fungal entomopathogens as paired microbial control agents. Enhancing our understanding of these roles will contribute to the promotion of EPF use for sustainable food production.

## 2. Mechanism of Fungal Endophytism in Plant Growth Promotion

Endophytic fungi can enhance agricultural productivity sustainably by improving nutrient uptake, producing plant hormones, and reducing ethylene levels. These mechanisms are key to boosting plant growth.

### 2.1. ACC Deaminase Production

Ethylene is a key plant hormone for fruit ripening, but too much may cause senescence and plant mortality. We may be able to decrease ethylene levels in stressed crops, which will result in higher yield in the unfavorable climates. Subsequently, numerous chemicals have been used to manage ethylene levels, and 1-aminocyclopropane-1-carboxylate (ACC) is among the most common. ACC is an intermediate to ethylene and is transformed to ethylene by the enzymes of plants when they are stressed. Ethylene levels above normal could stifle root and shoot growth and then result in plant senescence. Certain plant rhizosphere microorganisms can synthesize 1-aminocyclopropane-1-carboxylate deaminase as a way of neutralizing the detrimental effects of ethylene stress [8,9]. There are other endophytic fungi which have the ACC deaminase enzyme that has the ability to reduce the production of ethylene by converting ACC to α-ketobutyrate and ammonia in plants. By treating plants with these fungi, they can be aided in tolerating ethylene from phytopathogens [10,11].

### 2.2. IAA Production

A class of small molecules that are capable of causing quantitative changes in the growth reactions of plants is auxin [12,13,14]. Indole-3-acetic acid (IAA), the most widely existing auxin hormone, is vital for cell division, elongation, differentiation, fruit development, and phototropic responses [12,13,15]. There are studies to prove that a wide range of microbes, from bacteria to actinomycetes, filamentous fungi, yeast, and others, may be involved in IAA production in association with plants, but this research is still limited [16,17,18]. IAA production is usually through both the Trp (Tryptophan)-dependent and Trp-independent pathways, whereby some organisms may use a combination of the two, with the majority of studies focusing on the Trp-dependent pathways. In fungi, three distinct Trp-dependent biosynthetic pathways for IAA production have been identified: (IAM), (IPyA), and (TAM) are the products of indole-3-pyruvic acid (IPyA) and tryptamine (TAM), respectively [19,20]. Distinctive metabolic intermediates of every pathway have been found in different fungal species and this is one of the ways to differentiate them. The latest studies concerning the functions of fungal-derived IAA in plant–fungal interactions suggest that fungi can possibly use IAA to enhance their colonization of plant tissues and further stimulate plant growth and modulate plant defenses [21,22].

### 2.3. Phosphate Solubilization

Lower-concentration rock phosphate serves as the primary raw material for the formulation of phosphate mineral apatite [Ca_5_(PO4)_3_(Cl/F/OH)]. However, the production of usable phosphate from this source requires a high energy input, along with additional chemicals [23]. Microbial production of organic acids (OAs) with hydroxyl and carboxyl groups play a crucial role in solubilizing inorganic phosphorus (P) by chelating cations, primarily calcium, adjacent to phosphate, thereby converting it into soluble forms [24]. The efficacy of solubilization is determined by the number of carboxylic groups present. Many symbiotic fungi have the ability to produce citric and oxalic acids, which, as tri- and di-carboxylic acids, exhibit strong solubilization properties for inorganic phosphate, thus increasing phosphorus availability for plants [25,26,27,28,29]. Fungal acidification boosts plant phosphorus uptake by solubilizing less reactive phosphate sources and aiding in the release of P from soil minerals through processes like H+ extrusion and organic acid production. In the initial situation, phosphate-solubilizing fungi (PSF) have the potential to increase the efficacy of low-reactivity phosphate sources either through pre-treatment before soil application or by directly introducing them into fertilized soil. In the second instance, PSF can alleviate soil phosphorus fixation, leading to increased efficiency in fertilizer use and decreased demand for phosphorus fertilizers [30].

### 2.4. Zn Solubilization

Zinc (Zn) is one of the most important nutrients for plants in terms of their growth and sustainable crop production [31,32,33]. It is a coenzyme in more than 1200 proteins which include zinc-finger proteins, Zn-finger transcription factors, RNA polymerases, and DNA polymerases, and it is essential for processes such as the reconstruction of the PS-II complex during photo-inhibition, the maintenance of CO_2_ concentration in the mesophyll, hormone secretion, and MAPK-based signaling [34,35,36]. The microbial strains with zinc solubilization capacities, which are usually referred to as zinc-solubilizing microbes (ZSM), have become a hot topic in agriculture due to their ability to quickly solubilize the insoluble Zn compounds [zinc carbonate (ZnCO_3_), zinc sulfate (ZnS), and zinc oxide (ZnO)]. This microorganism produces organic acids that bind Zn^2+^ cations and change the pH of soil in the proximity of the plant root system [37]. Microbial processes like acidification, chelation, and chemical transformation play a key role in solubilizing zinc. Fungi produce organic acids like gallic acid, caffeic acid, and others to make insoluble zinc sources available for plants. For instance, Martino et al. (2003) [38] revealed that mycorrhizal fungi produced organic acids to convert insoluble zinc phosphate [Zn_3_(PO4)_2_] and zinc oxide [ZnO] into water-soluble Zn.

### 2.5. Nitrogen Uptake

Symbiotic fungi play a crucial role in enhancing the overall fitness of their plant partners by facilitating the uptake of essential nutrients, such as nitrogen [39], primarily by expanding the root surface area during colonization through mobilization [40,41]. Nitrogen, an essential nutrient for plant development, may be obtained from decomposing organic materials. Interestingly, a certain endophytic fungus can help plants absorb nitrogen from dead insects [42]. Numerous mycorrhizal species possess the capability to decompose organic debris in soil and deliver usable inorganic nitrogen to plant roots [43,44,45,46,47,48]. Mycorrhizal fungi may directly decompose the dead insects and their frass to access nitrogen that is eventually transferred to the plant by the translocation of nitrogen, sucrose, and minerals at the plant–fungi interface [49]. Indeed, it is worth noting that increased carbon supply to plants results in an intensified transfer of nutrients from the fungus to the plant, which once again highlights the vital role of carbon flow from the plant to the fungus in the process of nutrient transfer within plant–fungal symbioses [50]. Plants which are endowed with the ability of forming highly interactive relationships with nitrogen-fixing mycorrhizal fungi possess the tendency of outperforming their competitors in terms of nutrient acquisition from soil, in particular nitrogen, and this ends up in the achievement of high productivity and overall vitality of the plant.

### 2.6. Siderophore Production

Iron is the fourth most abundant transition metal present on the Earth’s crust after oxygen, silicon, and aluminum [51,52]. In this case, iron(III) and iron(II) oxidation states are equally feasible. In this case, it is not a secondary participant, rather it is a main participant, as it performs vital functions in oxidation–reduction processes. Furthermore, iron as a component of photosynthesis, oxidative phosphorylation, the electron transport chain, and the tricarboxylic acid cycle is not a standalone process, but rather it is also associated with several other metabolic activities [53,54]. Moreover, it is involved in the synthesis of many other essential compounds which include the aromatic compounds, nucleic acids, cytochromes, siderophores, vitamins, antibiotics, toxins, and pigments [53]. The etymology of the word siderophores is from Greek, sideros meaning “iron” and phores meaning “carrier”. Thus, they are agents that bind soluble ferric iron from a range of environments [55]. Though iron is a micronutrient, the deficiency of iron will hugely lower the quantity and quality of crop production. Siderophores specifically bind to iron, and that leads to a decrease in the amount of usable iron for the plant pathogens. In this way, the suppression of phytopathogens is facilitated [56,57]. They supply iron to plants when iron availabilities are limited, hence promoting plant growth [58]. Nevertheless, siderophore-mediated iron nutrition is still an enigma with gaps in the understanding of the mechanism. Two possible ways have been suggested for plants to obtain iron from microbial siderophores: (i) the transport system can counteract the high oxidation potential of microbial siderophores by supplying ferrous ions, and (ii) microbial ferric ions can penetrate the plant across the apoplast, where siderophores undergo reduction [59]. Consequently, an elevated concentration of ferrous (FeII) ions in the apoplast leads to the accumulation of Fe and Fe (II) in roots [60]. Microbially derived siderophores can additionally bind iron from soils and exchange ligands with phytosiderophores [61]. Increased volume of iron sequestration has been noted in plants that are associated with mycorrhizal fungi when compared to plants not associated with them, indicating that the enhanced nutritional support provided to plants by mycorrhizal fungi depends on the presence of fungal siderophores [62,63], which regulates biological systems by competing for Fe, thus decreasing the availability of Fe for plant pathogens [64,65,66].

## 3. Mechanism of Fungal Endophytism in Biocontrol

Endophytic fungi are a prominent class of microbial biocontrol agents (MBCAs) that fight phytopathogens and live on different sections of plants. Some of the defensive strategies they employ involve eliciting systemic responses, generating secondary compounds like lipopeptides, antibiotics, and enzymes that possess antifungal properties, and competing with detrimental microbes for resources. Unique genes found in endophytic entomopathogenic fungi cause the expression of defense mechanisms in the host plants, including lytic enzyme secretion, competitive exclusion, antibiosis, and siderophore production. Indirect defense mechanisms include induced systemic resistance (ISR) and systemic acquired resistance (SAR) [67].

### 3.1. Competition

Competition stands out as a pivotal mechanism utilized by endophytes to impede the colonization of host tissues by pathogens [68]. This entails the local or systemic infiltration of plant tissues, exploiting accessible nutrients, and occupying spatial niches, thereby establishing an environment unfavorable for pathogen proliferation [69,70]. Generally, the competitive prowess of endophytes is synergistically intertwined with other mechanisms rather than operating in isolation [68,71,72].

### 3.2. Mycoparasitism

This vital strategy employed by endophytes to preserve the host ecosystem involves directly confronting the identified pathogen or its propagules [73]. Endophytic fungi commonly employ a strategy wherein they infiltrate the hyphae of pathogenic fungi and disrupt the cell walls of pathogens by causing cell lysis. This mechanism can be delineated by four steps: the initial and secondary stages involve the directional growth of endophytic fungal mycelium towards pathogenic fungi to locate them through chemotropism, while the subsequent steps encompass close physical interaction between endophytic and pathogenic fungi, leading to the infiltration of the targeted fungal cells and subsequent degradation of the cell wall [74]. A prevalent form of antagonism involves the direct physical contact of mycoparasites with host hyphae, wherein the mycoparasites often coil around the host, penetrate it, absorb nutrients, and ultimately disintegrate the host cell [75,76]. This process is facilitated by prominent enzymes that degrade the cell wall such as chitinase, cellulase, xylanase, and glucanase [77,78].

### 3.3. Production of Secondary Metabolites for Antibiosis

Organisms have developed survival strategies, including the production of allelochemicals to inhibit competitors’ growth and neutralize toxic effects from nearby organisms [79,80]. Volatile organic compounds (VOCs) are secondary metabolites that play a significant role in the defense mechanisms of plants and microorganisms. They help protect against pests or pathogens and facilitate communication within or between species during attacks [79]. VOCs also assist organisms in surviving with limited resources in specific adverse niches and inhibiting competitors. Further investigation has indicated that the biosynthesis of hydroperoxy oxylipins, catalyzed by lipoxygenases (LOX), is upregulated during these interactions [81], potentially facilitating the adaptation of endophyte characteristics in the host to ecological changes [82,83]. Plants infected with endophytic fungi (EFs) exhibit a higher abundance of bioactive chemical compounds compared to those infected with pathogenic fungi, and they may also contribute to the transition from pathogens to endophytes and vice versa [84,85]. Epiphytes, pathogens, and host defense mechanisms compete with facultative EFs when they colonize host plants. This rivalry may explain why EF-infected plants generate more defensive compounds than pathogen-infected plants. Endophytic fungi may control host plant secondary metabolite (SM) synthesis, and several ecological stressors in natural or farmed contexts cause stress-specific transcriptional changes. In endophytic symbiosis, EFs modify the production of SMs in hosts by utilizing primary phytochemicals as precursors and secreting various protein effectors as signaling molecules [86,87,88]. Each effector exhibits specificity towards a distinct hormone signaling pathway [89].

### 3.4. Stimulation of Resistance

Endophytic fungi are renowned for their role in eliciting plant protection mechanisms that combat pest or pathogen assaults. This elicitation enhances the plant’s defensive reactions against subsequent pathogen attacks, facilitating improved performance; this phenomenon is also termed induced systemic resistance or priming defenses [90]. Induced systemic resistance (ISR) is typically modulated with the help of ethylene or jasmonic acid (JA), without the elevation of pathogenicity-related (PR) proteins [91]. Conversely, systemic acquired resistance (SAR) is frequently linked to pathogen infection and is controlled by salicylic acid, resulting in the augmentation of PR proteins [92], such as chitinase and beta 1,3-glucanase, that directly degrade pathogen cells and reinforce the cell wall barrier to resist infection and cell disintegration [93,94]. It is necessary to conduct further in-depth research to fully understand this process [95]. As there are microbial-derived substances that are readily identified by plant receptors, pathogen-associated molecular patterns (PAMPs) and microbial-associated molecular patterns (MAMPs) play important functions. ISR and SAR represent two forms of induced defense mechanisms triggered by beneficial and pathogenic microbes, respectively. With salicylic acid (SA) accumulation, along with a redox-regulated protein called non-expressor of pathogenesis-related genes 1 (NPR1), activated systemic acquired resistance (SAR) leads to the induction of pathogenesis-related (PR) genes [92]. In contrast, the jasmonic acid and ethylene (JA/ET) signaling pathway is linked with induced systemic resistance without the activation of PR genes [96,97,98] and is induced by beneficial microbes through a dependent pathway. However, recent findings suggest that multiple hormone signaling pathways might be implicated in shaping ISR [99,100,101], indirectly bolstering plant defenses and encompassing various defense strategies such as induction of antioxidant enzymes, callose deposition, enhancement of defense-related genes, phytohormones, and accumulation of transcription factors. Plants possess an innate immune system genetically encoded with a plethora of receptors, which recognize MAMPs and PAMPs as well as pathogen effectors (PAMPs, proteins, and metabolites) [102], designed to enhance pathogen invasion and, unfortunately, activate plant immune receptors [103]. The detection of MAMPs or PAMPs and damage-associated molecular patterns (DAMPs) outside the cells initiates the primary indispensable protection mechanisms known as pattern-triggered immunity (PTI) [104,105], playing a crucial role in protecting the host from infection by non-adapted microbes and also limiting the invasion of adapted pathogens in vulnerable plants. The plants’ initial defense mechanism involves detecting molecular patterns from microbes and the host, for which plants harbor a vast array of receptor-like kinases (RLKs) and receptor-like proteins (RLPs) acting as pattern recognition receptors (PRRs) [105]. MAMP-triggered immunity (MTI) is generated by PRRs. DAMPs stimulate another aspect of PTI after cellular breakdown. PRRs help innate immunity by recognizing DAMPs [106]. Additionally, upon assault, the effector kinase within the PRRs complex can become activated, resulting in an elevation in CNGC-mediated cytosolic calcium (Ca^2+^), a vital signal for PAMP-triggered immunity (PTI) in plants [107]. Pathogens use effector-triggered susceptibility to avoid PTI and infect plant cells. In response to effector-triggered susceptibility (ETS), plants develop a secondary immune system with enhanced effector-triggered immunity (ETI) inside the cell. So, PTI and ETI regulate ion movement across the plasma membrane and boost cytosolic Ca^2+^ and ROS levels in the apoplast, activating mitogen-activated protein kinase (MAPK) and leading to nitric oxide (NO) accumulation. This is succeeded by phytohormone production, closure of stomata, deposition of callose, and alterations in transcriptional and metabolic processes linked to defense [104,108,109].

### 3.5. Plant–Insect–Endophytes Tritrophic Interaction

The plants have developed a diversity of mechanisms to ensure the increase in the insect pest antagonist population, which act as protective agents, and the concept was refined by the bodyguard hypothesis [110]. The natural enemies of pests like insect parasitoids, predators, and entomopathogens (such as bacteria, viruses, and fungi) are quite impacted by volatile organic compounds (VOCs) released by healthy plants. These volatiles can suppress the conidiation of entomopathogenic fungi (EPF), while those emitted by herbivore-damaged plants may promote the growth of EPF, which can be observed in various plant parts including stems, leaves, and roots. Plants host a diverse array of endophytic fungi, encompassing both entomopathogenic and non-entomopathogenic species. This endophytic association often diminishes the growth and performance of polyphagous and sucking insects, although it has less impact on monophagous insects. It is believed that the adaptation of monophagous insects to secondary metabolites produced by fungi may account for this phenomenon. Herbivore or microbial attacks on plants induce alterations in plant molecular physiology and biochemistry, a phenomenon confirmed through gene expression and transcriptional reprogramming [111,112,113,114,115,116]. These alterations synchronize the production of volatile and non-volatile compounds, as well as the nutrient profile in plants, in addition to emitting optical signals for insects [111,117]. Microbes can influence or regulate insect fitness and behavior in tripartite interactions, known as tri-trophic interactions [118,119], which may impact insect behavior along with the spread of pathogen infections [120]. Understanding the relationship between plant microbes and insects is crucial from an eco-physiological perspective, facilitating genetically engineered improvements in crops and integrated pest management strategies that take into account changes in physiological conditions (such as age, mating, and feeding), thereby adapting their olfactory systems [121]. Moreover, microorganisms such as fungi and bacteria are capable of producing volatile organic compounds (VOCs) [122,123]. These VOCs can indirectly induce plant VOC production, leading to attraction or repellence between insects and plants, thereby influencing a cascade of behavioral responses associated with fitness-driven survival [120,124,125,126,127]. Many microbial VOCs are closely related to insect VOCs that stimulate feeding behaviors and oviposition [128]. VOCs produced by microorganisms often mimic phytohormones, as evidenced by the reduced attractiveness of insect larvae resulting in their evasion [129]. The attraction of insects based on VOCs from microbes or pathogen-infected plants depends on each organism involved in this interaction, and may alter host–herbivore and herbivore–predator interactions influenced by a wide array of signals between organisms, necessitating extensive research from an integrated and comprehensive perspective.

## 4. Methods of Inoculation

**Figure 1:**
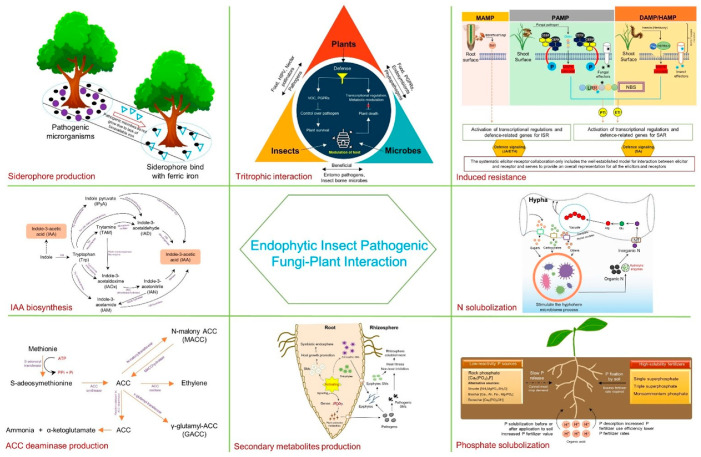
Main endophytic mechanisms towards plant growth promotion and biocontrol.

In addition to culture medium, endophytica microorganism domestication relies on inoculum density, fungal entomopathogen strains, targeted plant species, and culture conditions [130,131,132,133]. Studies show that the culture medium affects endophytic colonization more than inoculation strategy [134]. Tefera and Vidal (2009) found that *Beauveria* (Balsamo-Crivelli) Vuillemin (Hypocreales: Cordycipitaceae) may colonize sorghum leaves, stems, and roots without inoculation [135]. However, sterile soil, non-sterile soil, and vermiculite affect colonization differently. Other studies have shown that inoculation technique, fungal isolates, and plant types may affect *B. bassiana* endophytic colonization. Due to their absence of stomata, conidia are typically too large to enter green leaves [132,133]. Chemicals in leaf cuticular components may also limit conidia infiltration, thus lowering disease colonization [132,133]. Stem injection is one of the best ways to artificially inoculate beneficial insects like EPF instead of crop insects. This method skips the outer cell layer and other plant physical barriers, making it excellent for introducing conidia directly into plant tissues [132]. Soaking seeds in conidia suspension allows endophytic fungus to sexually reproduce and invade host plants [136]. Soil factors, both abiotic and biotic, have also been found to influence endophytic colonization, with biotic factors having a greater impact than abiotic factors. Poor endophytic colonization has been observed in non-sterile soil conditions [136]. Dipping seedling roots in conidial suspensions has been identified as another effective method for endophytic colonization of host plants. For instance, Russo et al. (2015) proposed that foliar inoculation may be a more suitable approach compared to root dipping for crops such as tobacco, corn, wheat, and soybean, as it resulted in a higher percentage of colonization by *B. bassiana* [137]. The success of artificial inoculation methods is greatly influenced by the plant species, as different species may respond differently to various inoculation techniques. Soil drenching, which entails applying conidial suspensions around the root area of seedlings, has proven effective for numerous crop plants [134,135,138] (Figure 1).

## 5. Recent Advances of “Endophytism” Exerted from Entomopathogenic Fungus

*Akanthomyces* spp. Lebet (Hypocreales: Cordycipitaceae) are recognized for their capability to effectively control specific pests and pathogens. Nevertheless, their ability to combat the pine processionary and certain phytopathogenic fungi such as *Fusarium* Link (Hypocreales: Nectriaceae) and *Curvularia* Boedjin (Pleosporales: Pleosporaceae) has yet to be investigated. *Akanthomyces muscarius* has shown great potential as a viable option for biocontrol against these pests and diseases. Strain TA01 showed remarkable inhibitory effects on the radial growth of different fungal strains, including four *Fusarium* strains and one *Curvularia* strain. The inhibition rates ranged from 39.61% to 52.94%. Considering the strong connection between *Akanthomyces* species and the *Verticillium* Nees (Glomerellales: Plectosphaerellaceae) genus, it is reasonable to assume that they have comparable mechanisms of action. For example, the mode of action of *Verticillium lecanii* may include chitinase activity [139] (Table 1).

Through rhizosphere inhabitation or endophytic establishment in root tissues, many entomopathogenic fungi such as *Metahizium* Sorokin (Hypocreales: Clavicipitaceae) and *B. bassiana* species are closely associated with plants. These characteristics augment the capacity of endophytic entomopathogenic fungi to persist within the root system when utilized as agents for biological control. The colonization of interior plant tissues by endophytic entomopathogenic fungi is also regulated by the plants themselves. This might be seen as a defensive strategy evolved by plants to repel herbivorous insects. On the other hand, the total amount of root endophytic colonization increased noticeably by 112% when *Fusarium graminearum* was present. Different isolates of *Metahizium anisopliae*, which were introduced to maize plants via seed coating and root herbivory induced by larvae, resulted in varying concentrations of salicylic acid (SA) and jasmonic acid (JA). *M. anisopliae* A1080-treated maize plants had more SA and JA in their roots than controls. On the contrary, when larvae were present, maize plants that were inoculated with *M. anisopliae* F672 had elevated levels of both phytohormones, suggesting that they may have been primed. *Metarhizium*-treated plants also show changes in SA and JA levels, which are connected to systemic acquired and induced resistance. Their findings demonstrated the important role that soil variables have in the interactions that entomopathogenic fungi have with plants, which in turn influences plant physiology. Two types of induced resistance are recognized: induced systemic resistance (ISR) and systemic acquired resistance (SAR). Micro-lesions produced by necrotizing infections set off SAR, which in turn causes the protein NPR1 to control the production of defense-related genes and the buildup of salicylic acid (SA) [140,141]. Rhizobacteria that promote plant growth are not pathogenic but rather cause ISR. It is not SA-dependent and is dependent on both NPR1 and the jasmonic acid/ethylene pathway [140,141].

Treating maize plants with *M. anisopliae* A1080 led to higher levels of salicylic acid (SA) compared to plants not treated or treated with *M. anisopliae* F672. This indicates that *M. anisopliae* A1080 has the potential to trigger SAR in the plant or be identified as a pathogenic organism. In contrast, maize plants treated with *M. anisopliae* F672 showed lower levels of SA, indicating that they may not see this strain as a threat. Additional research is needed to understand how the increase in SAR in the plants by *M. anisopliae* A1080 affects plant resistance to above-ground predators. The JA profile differed greatly between the two entomopathogenic fungal treatments. Maize plants treated with *M. anisopliae* A1080 produced more JA in roots and shoots than untreated plants, suggesting that *M. anisopliae* A1080 may cause ISR. Plants treated with *M. anisopliae* F672 showed the same levels of JA as control plants. Nonetheless, plants that received treatment with *M. anisopliae* A1080 showed the highest concentrations of this plant hormone in their roots and leaves. *M. anisopliae* A1080 demonstrates effectiveness in interacting with the phytohormones JA and SA by colonizing the rhizosphere and functioning as a root endophyte. The hormone profiles seen in the isolates may differ due to their sources: *M. anisopliae* A1080 was found in an insect larva, while *M. anisopliae* F672 was discovered in a *Pinus radiata* D. Don *(Pinales*: *Pinaceae*) root. The presence of *C. giveni* in control plants resulted in a significant rise in JA levels in leaves compared to control plants without the pest. There is strong evidence suggesting that JA signaling is mainly triggered in roots by root herbivory. Nevertheless, it is crucial to acknowledge that roots generally show a less noticeable JA reaction to herbivory when compared to leaves [142,143,144].

Entomopathogenic fungi have an impact on many systems that control plant disease resistance. However, the function of plant endophytic microorganisms in disease resistance is still not well understood. A prior study found that there was a significant reduction in the prevalence of northern corn leaf blight on maize when the soil was inoculated with *Beauveria bassiana*. Simultaneously, the introduction of *B. bassiana* and the infection produced by *Exserohilum turcicum* (Pass.) K.J. Leonard and Suggs (Pleosporales: Pleosporaceae) led to an increase in both the variety and number of endophytic bacteria and fungi, respectively. Network analysis demonstrated that the colonization of *B. bassiana* had a stronger impact on bacterial populations in comparison to fungal communities. The resistance to *E. turcicum* illnesses caused by *B. bassiana* may be associated with changes in the organization of the leaf endophytic microbial population and its network complexities [145].

Seeds were treated with spores from *Metarhizium* spp. or *Beauveria bassiana*, and their effect on seed germination and plant dry weight was assessed [146]. Furthermore, assessment of larval mortality and observation of symptoms linked to *Fusarium* root rot were conducted. The research showed that entomopathogenic fungi are consistently found in the soil around maize plants when using a specific *Metarhizium anisopliae* strain with green fluorescent protein expression. Both competitors’ presence negatively impacted the growth of maize, leading to a 33% decrease in root dry weight in treated plants. Nevertheless, there were no notable differences observed when seeds were treated with entomopathogenic fungi. Some seed coatings were found to be quite effective in decreasing *Fusarium* root rot symptoms by 24–44% and triggering a mycosis rate of up to 67% in *Costelytra giveni* Coca-Abia and Romero-Samper (Coleoptera: Scarabaeidae) larvae. This study highlighted the effectiveness of a seed coating with *Metarhizium* or *Beauveria* conidia for pest and plant pathogen control. At the same time, hyphae established strong links with plant roots after conidial sprouting. The study demonstrates that using entomopathogenic fungi on maize seeds can successfully protect against *C. giveni* and *F. graminearum* by living on maize roots. In spite of difficulties, certain strains of *Metarhizium* successfully improved the growth of maize plants. Variability is present in the efficacy of various entomopathogenic fungal strains in terms of controlling diseases and pests, as well as promoting plant growth. Therefore, it is essential to accurately characterize isolates and species of entomopathogenic fungi, as well as understand their interaction with the host plant and competitors [146].

*Metarhizium* spp. is an entomopathogenic fungus that, when grown in liquid media, may create large numbers of tough structures called microsclerotia. Microsclerotia are able to withstand drying out, stay stable in storage, and proliferate infectious conidia in vast numbers when rehydrated. On average, around 1 × 10^1^ microsclerotia/mL were formed by specific *Metarhizium robertsii* (F447) and *Metarhizium anisopliae* (A1080 and F672) isolates. These microsclerotia were made into granules by grinding them with diatomaceous earth and were then coated onto the seeds. This process yielded around 5 × 10^6^ CFU per gram of seeds. *Metarhizium* microsclerotia considerably increased plant growth compared to control plants, whereas untreated maize plants showed the lowest growth when exposed to the plant pathogen. Under all *Metarhizium* spp. treatments, fungal hyphae multiplied on and within the root tissues, whereas control plant samples showed no hyphae at all. The presence of *Metarhizium* fungus as endophytes was verified one month after sowing by observing hyphal penetration sites on the roots. The results of this study indicate that microsclerotia may be used to protect maize seedlings against soil-borne illnesses and transfer *Metarhizium* into the root zone of plants, a biocontrol agent that will remain in the soil for a long time. Near the nutrient-rich rhizosphere, the conidia are released and quickly germinate, eventually becoming hyphae. The symbiotic association between growing roots and hyphae that originate from microsclerotia or conidia is established. Because of their well-documented rapid growth and capacity to produce different types of fungal propagules, such as submerged conidia, blastospores, blastoconidia, and budding hyphal cells through microcycle conidiation, *Trichoderma* Pers. (*Hypocreales*: *Hypocreacea*) and *Beauveria* isolates have been found to have a higher biomass than *Metarhizium* [147].

*Aspergillus nomiae* Kurtzman, B.W. Horn and Hesselt (Eurotiales: Aspergillaceae), typically known as a pathogenic fungus affecting both humans and plants, has recently revealed potential beyond its conventional role [148]. An entomophagous fungus (EPF) strain, designated AnS1Gzl-1, was isolated and examined from *Spodoptera litura* Fabricius (Lepidoptera: Noctuidae) larvae found in a soybean field. This strain exhibited pathogenicity against various insect pests, focusing primarily on its ability to inhabit plants and induce resistance against both phytopathogens and insect pests. AnS1Gzl-1, identified as *A. nomiae*, displayed significant pathogenic effects on five insect pests belonging to the Lepidoptera and Hemiptera orders. Furthermore, it effectively suppressed the growth of *Sclerotinia sclerotiorum* (Lib.) de Bary (Helotiales: Sclerotiniaceae), a common soil-borne plant pathogen, when tested in vitro. Through root irrigation, plants were efficiently colonized by AnS1Gzl-1 as an endophyte, achieving a remarkable colonization rate of 90%. This colonization not only bolstered the plants’ immunity against phytopathogens but also disrupted the feeding habits of *S. litura* larvae. There are reports indicating that *A. nomiae* strains have the capacity to produce both series B and G aflatoxins [149], which are linked to the development of hepatocellular carcinoma, lung adenocarcinoma, and chronic inflammatory conditions [150]. Therefore, it becomes imperative to investigate in future studies whether toxins are metabolized by the *A. nomiae* strain AnS1Gzl-1 within its endophytic host plants. *B. bassiana* and *M. brunneum* exhibited a notable reduction in the expression of genes associated with plant immunity and the synthesis of hormones such as auxin, cytokinin, and gibberellin. Their findings were consistent with the zig-zag model of plant immune system activation [151,152].

Following the initial stages, the immune system response progresses to activate ETS through effectors released by thriving microorganisms. Consequently, this inhibits PTI. Plants then demonstrate ETI as a subsequent response. Effectors derived from *Beauveria bassiana* and *Metarhizium brunneum* Sorokin (Hypocreales: Clavicipitaceae) have been observed to impede the immune response, facilitating the colonization of plants by these organisms [153]. Moreover, this colonization process instigates systemic resistance [154,155,156]. The observed relative gene expression (RGE) patterns at 2 and 36 h post-infection (hpi) suggest a possible suppression of systemic acquired resistance (SAR) and activation of the jasmonic acid (JA) signaling pathway by additional microbial effectors [157,158]. Alternatively, the activation of the abscisic acid (ABA) signaling pathway, particularly noticeable with *B. bassiana* at 36 h post-infection (hpi), may potentially induce stomatal closure [153]. This mechanism could act as a strategy to impede further fungal colonization of the tissue. Hence, the production of secondary metabolites for bolstering the immune system represented a substantial investment that restrained plant growth [152,159,160,161].

Hence, the interplay between plants and fungi in controlled settings might exhibit consistent durations and patterns, resembling the zig-zag model, irrespective of the specific crop under investigation. Besides temporal changes, variations in gene expression patterns were noted among different wheat tissues. The growth of plants heavily relies on auxins, cytokinins (CKs), and gibberellins (GAs), pivotal substances that facilitate cell growth, division, and elongation. Utilizing *Beauveria bassiana* (NATURALIS) and *Metarhizium brunneum* (BIPESCO5) to treat seeds could establish these beneficial fungi within wheat plants, thereby enhancing plant growth. This study delves into the influence of fungal strains acting as endophytes on *Fusarium culmorum*, a primary culprit behind crown and root rot (CRR) in wheat. Both *B. bassiana* and *M. brunneum* exhibit the capability to colonize both the roots and shoots of wheat plants, leading to enhancements in various plant growth parameters, including shoot height, root length, and fresh root and shoot weights [162].

Root and shoot tissues of plants underwent systemic colonization at 14 and 24 days post-inoculation, subsequent to soaking wheat seeds in fungal inoculum of either *Beauveria bassiana* or *Metarhizium brunneum* for 16 h. Moreover, the study reveals that in conditions of limited nutrients, endophytic presence of *M. brunneum* notably enhanced various physiological parameters, including quantum yields of photosystem II, CO_2_ assimilation, stomatal conductance, and water usage efficiency. Interestingly, soil fertility did not influence the advantageous effects of *M. brunneum* endophytes on plant nutrition. In scenarios of scarce soil resources, fungal entomopathogens acting as endophytes confer greater benefits to plants compared to mycorrhizal–plant beneficial interactions [163]. As per the findings, in nutrient-deficient conditions, the fungus can function as a sink. Indeed, this finding fulfills the requirements for being classified as an endophyte [164]. The fungus must establish residence within the living tissues of plants, either throughout its entire lifecycle or for a portion of it, without inducing any discernible damage or symptomatic infection. Such phenomena may vary depending on the interplay between the fungal genotype and the plant genotype within different environmental contexts [2].

Larvae typically exhibit a preference for uncolonized leaves over those that have been colonized by *Beauveria bassiana*. This preference is supported by the noticeable decrease in the weight of the leaves preferred by the larvae. One possible explanation for this phenomenon is the presence of secondary metabolites in plants that possess antiherbivore properties. Thus, it is probable that the presence of inhibitory metabolites (antibiosis) produced by fungi is responsible for the formation of these antagonism zones. *B. bassiana* is renowned for its production of a variety of significant metabolites, some of which exhibit antifungal properties. These metabolites include bassianolide, bassianin, beauveriolide, bassiacridine, cyclosporine, oosporein, and beauvericin. Likewise, *Metarhizium* sp. has the capacity to produce a diverse array of secondary metabolites, including destruxins, swainsonines, serinocyclins, and cytochalasins [165]. The inoculation and establishment of entomopathogenic fungi within plants emerges as an alternative and promising approach to mitigate the limitations imposed by environmental conditions in pest control efforts [166].

Possessing a fungal strain that can efficiently combat both pests and pathogens for plants might provide a significant benefit in crop improvement. Researchers examined how effective the entomopathogenic fungus *Metarhizium anisopliae* is in fighting against *Botrytis cinerea* Pers. (Helotiales: Sclerotiniaceae), the fungus responsible for gray mold disease which causes major economic damage. The assessment centered on *M. anisopliae’s* capacity to fight in response to the gray mold pathogen *Beauveria cinerea*. During the dual-culture test, a significant inhibition zone was observed in the presence of the *B. cinerea* colony caused by *M. anisopliae*. Results demonstrated that *M. anisopliae* may be a viable option for managing gray mold infestation for tomatoes in a greenhouse, even after harvest. Transmission electron microscopy showed notable harm to the cell organelles of *B. cinerea* mycelia caused by the *M. anisopliae*, indicating the release of a toxic substance by *M. anisopliae* affecting *B. cinerea*. Additionally, it is proposed that specific secondary compounds created by *M. anisopliae* could potentially affect the integrity of conidia’s plasma membrane, as shown via fluorescence emissions seen in propidium iodide-treated conidia. *M. anisopliae* was discovered to produce additional compounds, such as destruxins, swainsonine, and polyketides [167].

Four strains—Bb74040, Bb762, Bb716, and Bb688—demonstrated a notable reduction in symptoms caused by *Botrytis cinerea* and *Alternaria alternata* (Fr.) Keissl. (Pleosporales: Pleosporaceae) in living organisms. The infection rate decreased by 32 to 40% and 21 to 41%, respectively, while the necrotic area decreased by 35% to 73% and 45% to 63%. Notably, the study marks the first documented evidence of endophytic *B. bassiana* biocontrol against *A. alternata*. Indications of ISR were observed through a decrease in disease occurrence in plant portions distant from the site where the beneficial substance was introduced, as demonstrated in the bioassay described. Therefore, it may be hypothesized that either one of several indirect modes of action or a synergistic combination is responsible for the observed biocontrol activity. According to recent research, tomato plants harboring endophytes in their roots exhibit increased resistance. This is attributed to the activation of their defense mechanisms, characterized by the upregulation of specific transcripts encoding proteins associated with ET, JA, SA, and PR proteins. These proteins play a pivotal role in priming the plants for heightened defense, akin to what is observed in ISR and SAR [168].

Only a few studies have uncovered how plants defend themselves from invasion by *M. robertsii*. Genes that work in the JA biosynthesis pathway including lox1 and opr7 were found to be upregulated. On the other hand, *maize proteinase inhibitor* (*mpi*) expression was reduced in the leaf tissue of maize plants grown from *Metarhizium robertsii*-inoculated seeds compared to those grown from control seeds. It would be possible that the over-expression of the JA biosynthesis pathway in *M. robertsii*-inoculated maize plants, even in the absence of insect feeding, is the result of the high amount of JA precursors accumulating as a priming effect. The downregulation of mpi, a marker located more downstream, might be a sign of the ability of maize plants to react to the absence of external environmental factors such as insect feeding. Also, gene *pr5*, which is part of the SA pathway, showed upregulation in leaf tissue of plants originating from *M. robertsii*-inoculated seeds. The success of the SA-dependent defense response pathway in combating biotrophic pathogens and sap-feeding insects could be due to the fact that the plant perceived *M. robertsii* as a biotrophic pathogen [169].

Moreover, in conjunction with the development of other advanced tactics, maize plants harbor defensive proteins called endochitinase A and pr4. These proteins inhibit herbivores and promote pathogen defense [170]. Compared to controls, *M. robertsii*-inoculated seeds elevated endochitinase A and decreased pr4. The presence of chitin-degrading proteins, facilitated by the regulation of gene expression, can offer an additional layer of protection, even in the absence of herbivores. Leaf tissue of colonized plants showed changed plant defense genes. Endophytes reduced black cutworm larvae development on leaves relative to controls in feeding bioassays. Little is known about how plants respond to their colonization by *Metarhizium* spp. Nonetheless, the colonization of maize plants by *M. robertsii* exerted a beneficial effect on plant growth, eliciting changes in defense-responsive gene expression and restraining the growth of black cutworm larvae.

Furthermore, the performance of *Phthorimaea absoluta* (Meyrick) (Lepidoptera: Gelechiidae) on plants inoculated with entomopathogenic fungi (EPF) was assessed using the age-stage two-sex life table method [171,172]. Tomato plants exhibited accelerated growth and increased abundance following colonization with any of the tested fungi. Notably, *M. anisopliae* proved to be the most effective in enhancing biomass across all tissues. Regarding pathogenicity against *P. absoluta*, exposure to conidia of *Metarhizium anisopliae* and *Cordyceps fumosorosea* (L.) (Fr.) (Hypocreales: Cordycipitaceae) resulted in the highest levels, followed by *Beauveria bassiana* and *Metarhizium flavoviride*, while the lowest pathogenicity was observed when exposed to *M. rileyi*. Research has indicated that herbivorous insects may encounter antagonistic effects due to defensive chemicals induced in plants by endophytic entomopathogenic fungi. These compounds encompass monoterpenes, sesquiterpenes, benzoxazinoids, and flavonoids [173,174]. Further research has demonstrated that the introduction of entomopathogenic fungi exerts a counteractive impact on herbivorous pests by releasing toxins such as the cyclic peptides beauvericin, brassinolide, and destruxin. These toxins, released during endophytic colonization, serve to protect plants against herbivores [175,176,177]. The precise mechanism by which the five entomopathogenic fungi (EPFs) studied here influence the herbivore defense of tomato plants requires further clarification. Studies have demonstrated that fungi exhibit greater acid tolerance and possess the capability to mobilize specific bound phosphates in comparison to bacteria [178]. Many fungi have been documented to produce siderophores [179], as well as plant growth hormones such as indole-3-acetic acid (IAA) [180] and gibberellins [181]. A fungus known as *Lecanicillium palliate* W. Gams and Zare (Hypocreales: Cordycipitaceae) strain IISR-EPF-02, which has been observed to infect cardamom thrips *Sciothrips cardamomi* Ramakrishna (Thysanoptera: Thripidae), was discovered to enhance the growth of cardamom plants *Elettaria cardamomum* (L.) Maton (Zingiberales: Zingiberaceae). This particular isolate demonstrated its capacity to enhance plant development through the production of indole-3-acetic acid and ammonia, as well as by making an inorganic phosphate and zinc solvent. Additionally, it showed traits that enhance plant growth by producing siderophores and enzymes that break down cell walls, such as α-amylases, cellulases, and proteases.

During the *Arabidopsis*–*Metarhizium* split interaction, terpenes were the only compounds identified. Specifically, the presence of SQT β-caryophyllene was observed when the Ma-20 and Ma-25 strains interacted with *Arabidopsis* in the split system. However, when the Ma-28 strain was involved, only small amounts of β-caryophyllene and p-cymene were detected, with the majority of the mixture being the monoterpene o-cymene. Several terpenes, including β-caryophyllene and o-cymene, play significant roles in plants. They act as phytoalexins for plant defense or as signals in defense responses against herbivores and their natural enemies [182,183,184]. Three *Metarhizium* strains (Ma-20, Ma-25, and Ma-28), grown on MS media without plants, produced four volatile organic compounds (VOCs). Ma-25 and Ma-28 strains released β-caryophyllene, valencene, and a lesser quantity of 2-ethyl hexanol as sesquiterpenes (SQTs). The Ma-20 strain generated o-cymene, β-caryophyllene, and valencene in lesser amounts. These studies illustrate that each *Metarhizium* strain alters its VOC profile when interacting with the plant, potentially benefiting *Arabidopsis* plant development. Arabidopsis fresh weight and total chlorophyll significantly increased seven days post-inoculation with the three *Metarhizium anisopliae* strains.

The fungal genera *Metarhizium* and *Beauveria* display multipurpose traits, serving as both entomopathogens and endophytes. They possess the remarkable capability to establish themselves within a diverse range of plants, thereby promoting plant growth and offering protection against pests. Since the necessity of developing new biological methods for plant protection is evident, the present study was made to examine the effectiveness of two isolates of *Metarhizium robertsii* (ESALQ 1622) and *Beauveria bassiana* (ESALQ 3375) in suppressing the population growth of the spider mite *Tetranychus urticae* C.L.Koch (Trombidiformes: Tetranychidae) and improving the growth of bean plants. The research also examined the predatory mite *Phytoseiulus persimilis* Evans (Mesostigmata: Phytoseiidae) behavior and predation rates on fungal-treated plants and spider mites. Leaf disc experiments assessed the possible contradictory effects of fungal inoculations on higher-trophic-level pest control. The benefits of co-inoculants were tested by seed inoculation with *M. robertsii* and *B. bassiana* isolates alone and in combination. The research found that *M. robertsii* and *B. bassiana* alone and together reduced *T. urticae* numbers and increased plant growth. Despite the fact that the predation rate of the predatory mite *P. persimilis* on *T. urticae* from treated and untreated plants was similar, predators exhibited a preference for consuming spider mites from fungi-treated plants in the initial phase of the experiment, and spider mites from control plants in the latter phase. In general, the two fungal isolates show promise as seed inoculants for controlling spider mites in bean plants, and this approach appears to be compatible with the use of predatory mites. Additionally, *Metarhizium* spp. facilitates the transfer of nitrogen from infected insects to plants through mycelium–root connections in a tritrophic association involving host insects, fungi, and plants in the rhizosphere [185,186,187], thereby increasing overall plant productivity.

During stress, the endophytic partnership prompts the gathering and transmission of diverse secondary metabolites [188]. Isoflavones (IF), which are naturally synthesized by plants in reaction to various stressors like pests or environmental conditions, exemplify this phenomenon. Fungi, similarly, produce a broad spectrum of secondary metabolites, encompassing phytohormones (auxins, gibberellins, cytokinins, and abscisic acid), along with substances boasting antifungal and antibacterial characteristics [189]. *Metarhizium anisopliae*, an insect-pathogenic fungus, is renowned for its adeptness in outcompeting other organisms within the soil. It serves as a pivotal asset in pest management, particularly targeting insects during their soil-dwelling phase. Moreover, this fungus harbors the potential to function as a bioinoculant, aiding plants in mitigating abiotic stressors such as salinity by fostering their growth.

The combination of *Metarhizium anisopliae* (AAUBC-M15) and *Metarhizium pinghaense* (AAUBC-M26) on tomato plants without NaCl had improved results [190]. However, salt stress from 100 to 200 mM NaCl substantially decreased phosphate solubilization activity, except for co-inoculation at 100 mM NaCl. NaCl may reduce phosphate solubilization by inhibiting phosphatase enzyme activity or *Metarhizium* isolates’ organic acid synthesis. As plant probiotics, beneficial fungal isolates that generate ACC deaminase help plants tolerate biotic and abiotic stressors. Many fungi possess the capacity to produce chitinases, which can directly and indirectly enhance plant growth. Evidence shows that *Metarhizium* isolates produce phenolics and flavonoids in their culture filtrate. Exposure to 100 mM NaCl led to a significant rise in the levels of phenolics and flavonoids. Plants increase their phenolic production in response to unfavorable conditions as a defense mechanism. Accumulation of phenolics in higher plants in reaction to biotic and abiotic stresses is essential for their proper growth. Flavonoids act as powerful antioxidants to help plants thrive in difficult environments and promote growth. The inoculated fungus likely prompts plants to increase their production of phenolics and flavonoids, which helps detoxify reactive oxygen species (ROS) produced during times of elevated salt stress. Hamayun et al. [191] found similar results, stating that *Aspergillus flavus* Link (Eurotiales: Aspergillaceae) fungus also generates phenolics and flavonoids in its culture filtrates.

More than half of the larvae (80 to 90%) were infected by three different species of *Metarhizium* and *Beauveria*, whereas no other fungi efficiently infected more than half of the insects (47 to 50%) [192]. Moreover, the pathogens identified in the leaf fodder exhibited various plant growth-promoting (PGP) characteristics, including the capacity to solubilize organic and inorganic phosphates (104.7–236.4 μg/mL); produce siderophores, ammonia, and hydrogen cyanide (HCN); synthesize indole, and more. The genetic diversity and strain identification of potent biocontrol agents such as *Beauveria* and *Metarhizium* spp. were determined using techniques such as restriction fragment length polymorphism (RFLP), random amplified polymorphic DNA (RAPD), simple sequence repeat (SSR), and internal transcribed spacer (ITS) analysis. A positive correlation between phosphate solubilization (PS) and organic acid (OA) production was observed. Additionally, the varying intensity of reddening and mineralization zones in the phytate solubilization by entomopathogenic pungi (EPF) suggests qualitative and quantitative differences in their organic acid production [193,194,195]. Furthermore, in the initial stages of development, the fungus typically utilizes some soluble phosphorus (P), thereby mitigating the accumulation of P. However, as growth advances, the consumption of P slows down or halts entirely, and aging cells undergo lysis, releasing P. This phenomenon may contribute to an increase in the overall P level in the medium, particularly in older cultures. The production of additional enzymes, such as extracellular amylase, cellulase, protease, chitinase, pectinase, lipase, and lecithinase, provides supporting evidence for the pathogenic or virulent nature of the entomopathogenic fungus (EPF) [196,197], through the selective lysis of cell wall and/or membrane components, such as chitin, chitosan, structural proteins (mannoproteins and glycoproteins), lipids, and others, achieved by hydrolyzing α-1,4/β-1,4/β-1,3 glycosidic or peptide or ester linkages, the process suppresses pests as well as fungal and bacterial diseases [198,199]. The fungus’ capacity to produce indole-3-acetic acid (IAA) could yield several advantageous effects, including the regulation of plant development, inhibition of the rice blast disease caused by *Pyricularia oryzae* Cavara (Magnaporthales: Magnaporthaceae), and prevention of mycelial proliferation [198,199,200,201]. The use of siderophores would boost the availability of Fe^3+^, while the addition of NH_3_ would increase the availability of N and stimulate plant development [198,199,200].

Root drenching, foliar spray, and seed treatment represent potential methods for introducing endophytes [202], which, in turn, produce metabolites such as alkaloids, flavonoids, and terpenoids as a defense mechanism against microbes and pests [2,203,204,205,206]. The transfer of chemicals between the host and endophyte’s metabolic pathways enables the essential metabolic functions for their symbiotic relationship to flourish [207]. Enzymes that break down insects’ outer layers, strain genetics, and gene activation impact the insect-killing fungi’s virulence. The *Vitis vinifera* (L.) (Vitales: Vitaceae) plant contains many endophytes, which may survive within it for years. The fungus *Beauveria bassiana* has been the subject of thorough research among these endophytes. It demonstrates the capability to colonize a wide range of plants through various methods and in different plant regions. These findings emphasized the impact of the endophytic fungus *B. bassiana* on the growth of *V. vinifera*. Furthermore, the fungus was successfully re-isolated from various parts of the plant using the bait method. In summary, this investigation highlights the proficiency of *B. bassiana* in establishing itself within *V. vinifera* plants. It contributes to our understanding of its ability to function as an endophyte and underscores its positive impact on the root growth of plants.

A recent study discovered that the application of a fungal suspension containing *Isaria javanica* Frieder. and Bally (Hypocreales: Cordycipitaceae) and *Purpureocillium lilacinum* (Thom) Luangsa-ard, Hou-braken, Hywel-Jones, and Samson (Hypocreales: Ophiocordycipitaceae) enabled the retrieval of tomato seeds from various plant tissues (root, stem, and leaf) for up to 60 days post-inoculation [208]. Both endophytic isolates also induced significant mortality among adult *B. tabaci* on seedlings inoculated with *I. javanica* (51.92 ± 4.78%) and *P. lilacinum* (45.32 ± 0.20%) compared to the control treatment (19.29 ± 2.35%). Notably, control treatments exhibited significantly higher adult emergence rates (57.50 ± 2.66%) compared to treatments with *I. javanica* (15.00 ± 1.47%) and *P. lilacinum* (28.75 ± 4.78%). These findings provide compelling evidence of the promising biocontrol properties exhibited by endophytic isolates of *I. javanica* and *P. lilacinum* against whiteflies. Furthermore, these isolates demonstrate potential as plant growth promoters, suggesting new avenues for exploration. Total chlorophyll content (TCHL) measurements conducted weekly served as a reliable indicator of plant strength and health. Treated cotton plants displayed significant improvements in growth parameters compared to untreated ones, such as a rise in leaf and shoot number, apical bud count, plant height, stem thickness, and both fresh and dry biomass, as well as TCHL levels. In addition to monitoring plant growth, the study observed the infestation of *Aphis gossypii* Glover (Hemiptera: Aphididae) over six weeks. Notably, by the fifth week, a noticeable decrease in the number of aphids was observed on the inoculated plants compared to the control group. The evaluation highlighted the significant impact of *B. bassiana* coating treatments on both biostimulation and bioinsecticidal properties. Additionally, endophytes exhibited the ability to modify the chemical profile of plants by altering the composition of phytosterols, while also competing with insects for nutrients [209].

To provide a worthwhile alternative, researchers are actively studying several kinds of fungus for their possible use as beneficial plant bioinoculants. To find out how well *Purpureocillium lilacinum*, *Purpureocillium lavendulum*, and *Metarhizium marquandii* boost plant development, an evaluation was completed. The investigation was conducted to assess their viability as bioinoculants for the cultivation of soybeans, maize, and beans and also examined strains of *Metarhizium marquandii* and *Purpureocillium* spp. that were isolated from soil samples for their ability to solubilize phosphate (P) and to create indole acetic acid (IAA). Subsequently, the most promising strains were tested under greenhouse conditions on maize, bean, and soybean plants. The evaluation encompassed growth promotion parameters such as plant height, dry mass, and levels of phosphorus (P) and nitrogen (N) in both plants and the soil surrounding the roots. Interestingly, some strains exhibited the ability to enhance the availability of P and N, which is not commonly observed in fungi of this type. Furthermore, it was noted that the changes anticipated based on in vitro analysis were not consistently observed in plants [210].

Employing *Beauveria bassiana* for seed treatment in maize holds significant promise for crop management, offering the potential to enhance growth and provide protection against pests, particularly in nutrient-deficient conditions. In this context, the fungus assumes a pivotal role by assimilating a substantial portion of the plant’s photosynthetic products. In environments lacking sufficient nutrients, the competition for the allocation of resources between plants and fungi can significantly impact plant–herbivore interactions. This is due to the fact that the nutritional value of plants for herbivores can be influenced by the availability of soil nutrients [211]. Indeed, further research is essential to delve into the mechanisms by which *B. bassiana* promotes plant growth. The findings indicate that the relationship between *B. bassiana* and maize can be antagonistic or mutually beneficial and is contingent upon the nutrient levels in the soil. This underscores the intricate, contradictory, and context-specific nature of the plant–fungus interaction. Some argue that existing literature on plant–fungus interactions inadequately addresses the substantial variations that occur in agricultural settings as it often overlooks the influence of abiotic environmental factors.

*Isaria javanica* pf185, a highly esteemed entomopathogenic fungus, shows considerable promise as an agricultural biocontrol agent. However, its impact on plant growth remains uncertain. *I. javanica* pf185’s principal volatile components were located by combining gas chromatography-mass spectrometry with solid-phase micro-extraction. Three hexanones, heptane, 2,4-dimethylhexane, and two nonanones are among these substances. Based on these results, *I. javanica* pf185 may be able to protect plants from insects and illnesses while also improving their development. Further research is necessary to gain a deeper understanding of the mechanisms underlying the plant growth-promoting effects of *I. javanica* pf185 [212]. The fungal recovery rate (FRR) confirmed that both *Beauveria bassiana* and *Metarhizium anisopliae* were capable of colonizing maize tissues as endophytes. Therefore, the two chosen entomopathogenic fungal strains effectively established endophytic colonization instead of rhizospheric colonization in maize. Typically, biological functions are influenced by population density. Quantitative detection of endophytic fungi in plant tissues is necessary to fully understand the relationship between plant growth promotion and population density of these fungi. Further exploration is required to fully understand the plant growth-promotion mechanism in the interaction system between entomopathogenic fungi and plants. The entomopathogenic fungi show immense promise in both pest control and promoting plant growth, offering a fresh perspective on the ecological dynamics between plants, insect pests, and these fungi [213].

**Table 1:** Some recent findings of endophytism from entomopathogenic fungus.

SL No.	Fungal Strain	Acts on	Inoculation Method	Mode of Action	Endophytic Traits	References
1	*Akanthomyces muscarius*	Pine processionary	Spraying onto larvae	Dual biocontrol	Mycelial growth, spore production, and inhibition of fungal growth.	[139]
2	*Metarhizium* spp.	Maize roots and shoots	Pre-coating the seeds	Biocontrol	SA and JA increased.	[214]
3	*Beauveria passion*	Maize	Soil drench	Biocontrol	Antibiosis, competition, or parasitism by producing fungal secondary metabolites.	[145]
4	*Metarhizium* spp.	Maize	Seed coating	Biocontrol	Hyphae would survive on the exudates produced by plant roots.	[146]
5	*Aspergillus nomiae*	Soybean	Root irrigation with conidial suspension	Dual biocontrol	Regulating salicylic acid (SA) and jasmonic acid (JA) pathways.	[148]
6	*Metarhizium* spp.	Maize	Coated onto seeds	Biocontrol	Production of microsclerotia.	[147]
7	*Beauveria bassiana* and *Metarhizium brunneum*	Wheat	Coated onto seeds	Biocontrol	Repression of genes that regulate the biosynthesis of hormones like auxin, gibberellin, and cytokinin.	[215]
8	*Metarhizium brunneum*	Wheat	Seed soaking with conidial suspension	Mycoinsecticides	Induction of systemic plant resistance or secondary metabolites and promotion of plant growth.	[162]
9	*Beauveria bassiana*	Soybean	Foliar spray	Antifungal activity and antiherbivore effects	Inhibited mycelial growth, generation of an inhibition zone, and secondary metabolites produced.	[216]
10	*Metarhizium anisopliae*	Tomato	Coated onto leaves and fruits	Biocontrol	Inhibition zone generation, secondary metabolites are produced, and inhibition of mycelium growth.	[217]
11	*Beauveria bassiana*	Tomato	Seedlings were watered with conidial suspension	Dual biocontrol	Limiting of mycelial growth, the presence of a clear zone of growth inhibition, metabolite production, and induction of plant resistance via ISR or SAR.	[218]
12	*Beauveria bassiana*	Potato	Potato tubers were immersed in the conidial suspension	Biocontrol	Prevention of full-fledged sclerotium formation, production of an antifungal peptide, secondary metabolites, and increase in peroxidase activity.	[219]
13	*Beauveria bassiana*, *Metarhizium anisopliae*	Maize	Conidia suspension was used with hydroponic solution	Biopesticides and biofertilizers.	Realization of the fungal endophytic function.	[213]
14	*Lecanicillium psalliotae*	Cardamom	Application of fungus at the root zone of seedlings	Biopesticides and biofertilizers.	Production of indole-3-acetic acid and ammonia by solubilizing inorganic phosphate and zinc, producing siderophores, and cell wall-degrading enzymes.	[220]
15	*Metarhizium robertsii*	Maize	Coated onto seeds	Biocontrol	The upregulation of the JA and SA biosynthesis pathway and expression of endochitinase A was upregulated.	[221]
16	*Metarhizium robertsii*, *Beauveria bassiana*	Bean	Seed inoculation	Biocontrol	Production of secondary plant metabolites and induction of proteins related to plant defense.	[222]
17	*Metarhizium anisopliae*	Arabidopsis, tomato, and maize	Seedlings were inoculated with conidial suspension	Insect biocontrol and plant growth promotion	Production of secondary plant metabolites and volatile compounds.	[223]
18	*Metarhizium anisopliae* (AAUBC-M15) and *Metarhizium pinghaense* (AAUBC-M26)	Tomato	Soil application, seedling root dip, and foliar spray	Bioinoculant	Activation of ACC deaminase enzymes and increased chitinase enzyme activity. Total phenolics and flavonoids increased.	[190]
19	*Beauveria* and *Metarhizium* spp.	Rice	Spray at pre-flowering stage	Growth promotion and biocontrol	Organic/inorganic phosphate solubilization; siderophore, ammonia, hydrogen cyanide (HCN), and indole production; etc.	[224]
20	*Beauveria bassiana*	Grape vine	Foliar spray	Growth promotion and biocontrol	Antagonism, induction of plant host defenses, and host plant tolerance. Production of secondary plant metabolites.	[225]
21	Endophytic fungi	Wheat	Seeds were soaked in fungal spore supension	Growth promotion and biocontrol	Indole-3-acetic acid [IAA] production, phosphate solubilization, siderophore production, and increased hydrolytic enzyme production.	[226]
22	*Isaria javanica* and *Purpureocillium lilacinum*	Tomato seeds	Seeds were soaked in fungal spore supension	Growth promotion and biocontrol	Colonization endophytically.	[208]
23	*Beauveria bassiana*	Cotton	Coating onto seeds	Biostimulatory and bioinsecticidal	Modification of plants’ chemical profile by altering phytosterol composition, and they also compete with insects for nutrients.	[227]
24	*Purpureocillium lilacinum* and *Metarhizium marquandii*	Maize, bean, and soybean plants	Inoculation was carried out with suspensions of each fungal strain	Growth promotion and biocontrol	Solubilize phosphorus (P) and produce indoleacetic acid (IAA).	[210]
25	*Metarhizium flavoviride*, *M. anisopliae*, *M. rileyi*, *Cordyceps fumosorosea* and *Beauveria bassiana*	Tomato	Seed inoculation, root dipping, and foliar spray	Growth promotion and biocontrol	Colonization endophytically.	[228]
26	*Fusarium tricinctum* and *Alternaria alternata* RSF-6L	Rice plants	Foliar spray	Growth promotion and biocontrol	Production of indole acetic acid.	[229]
27	*Beauveria bassiana*	Maize	Seeds were soaked in conidial suspension	Growth promotion and biocontrol	It works where nutrients are abundantly available.	[230]
28	*Isaria javanica* pf185	Tobacco	Drench method	Growth promotion and biocontrol	Increased fungal volatiles.	[212]
29	*Beauveria bassiana*	Cayenne pepper plants	Spraying the suspension on the whole plant	Biofertilizer and biostimulant	Increase in the solubility index of phosphate and production of IAA.	[231]

## 6. Conclusions and Future Perspective

The traditional culture-based methods alone may not offer a comprehensive understanding of the diversity and taxonomic composition of fungal endophyte communities. Recently, advanced molecular tools have gained popularity, replacing traditional selective media methods. Techniques like restriction fragment length polymorphism (RFLP) enable the identification of individuals based on unique DNA cutting patterns via restriction enzymes in specific regions. Additionally, culture-independent techniques are commonly employed in this field. Alongside morphological characterization, molecular analysis has become a valuable tool for identifying and classifying fungi. However, challenges persist, including limited access to functional gene annotations in gene libraries of studied genomes. Various factors such as climatic conditions, vegetation and soil types, geographical location, and human and biotic activities significantly impact soil microbial communities and the effectiveness of entomopathogenic fungi (EPF) in open fields. The distribution and diversity of EPF in soils vary due to geographic and climatic factors. Most studies on EPF/endophytes have been conducted in controlled laboratory or greenhouse settings, with few field trials. Some studies have reported a lower success rate of endophytic colonization in plants treated with EPF in natural field soils compared to sterile soils.

Exploring the ecological diversity and adaptability of fungi is crucial for developing highly effective biocontrol strategies. The impact of EPF on natural enemies of insect pests and non-target organisms is a major consideration. Certain endophytic fungi can affect the toxicity of various poisonous substances, resulting in significant financial losses. Efficient plant colonization by EPF is influenced by factors such as the number, variety, spread, and health of the endophytes. Different inoculation methods are being explored to enhance colonization of multiple fungal strains, aiming for effective management strategies. Advancements in gene modification and RNA interference (RNAi) technology offer new possibilities for enhancing fungal strains’ virulence and overall performance. However, there are concerns regarding the potential opportunistic pathogenicity of some endophytic fungi in plants, animals, and humans. Increased government investment in biological control projects, focusing on classical, inoculative, and conservation strategies, is necessary. It is crucial to consider various inoculation methods and the host plant species for successful EPF integration into insect pest management programs. Employing a combination of culture-dependent and culture-independent techniques improves the detection of fungal entomopathogens colonizing plants. Understanding ecological factors is essential for harnessing the endophytic potentials of EPF effectively. Overall, foliar and stem injection methods tend to be more successful compared to seed, root, and soil inoculation methods, with host plant species choice playing a crucial role. In the future, introducing toxic genes into fungi using “vaccinated seeds or seedlings” with endophytic fungi may be necessary.

## Data Availability

Not applicable.
